# *Trypanosoma cruzi* and Chagas disease: diversity, progress and challenges

**DOI:** 10.1590/0074-02760210193chgsb

**Published:** 2022-05-06

**Authors:** Michael A Miles

**Affiliations:** 1 London School of Hygiene and Tropical Medicine

Bianca Zingales and Daniella Bartholomeu have produced a high-quality review on *Trypanosoma cruzi* and Chagas disease. Here, I comment on the progressive understanding of *T. cruzi* diversity, impact, and challenges.

*Diversity* - The wide-ranging diversity of *T. cruzi*, the agent of Chagas disease, became clear when multilocus enzyme electrophoresis (MLEE) analysis of human genetics was transposed to trypanosomatids, initially to *Trypanosoma brucei*, and then to *T. cruzi*. The application of MLEE to a rural area of Brazil, published in 1977, revealed two radically distinct genetic groups of *T. cruzi*, one associated with domestic transmission by the triatomine *Panstrongylus megistus* and the other with sylvatic transmission by *Triatoma tibiamaculata*.^1^ Strikingly, comparisons of *T. cruzi* MLEE diversity in Venezuela, published in 1981, revealed that the principal domestic disease agent in Venezuela corresponded to the prevalent sylvatic *T. cruzi* in the rural region of Brazil.^2^ This led to the hypothesis that different clinical presentations of Chagas disease in Brazil and Venezuela might be associated with the two distinct MLEE ‘zymodemes’ of *T. cruzi*.^2^

As indicated, in the detailed review of Messenger et al.,^3^ there followed a series of wide-ranging MLEE analyses of *T. cruzi* strains, which included the Amazon region (1978,1981,1984) Bolivia (1983, with Michel Tibayrenc), Chile (1984,1987, with Werner Apt), Paraguay (1984), Colombia (1985), Ecuador (1985), Central Brazil (1986). An outcome of this research was the identification and distribution of six different MLEE groups of *T. cruzi*, as summarised in Table I of the review of Messenger et al.,^3^ and with some evidence of hybrid isoenzyme profiles.

The advent of multilocus sequence typing (MLST), led to a more robust and direct genetic analysis of *T. cruzi* isolates. Souto et al.,^4^ cited in the Zingales and Bartholomeu review, used MLST to confirm presence of the same two principal genetic groups of *T. cruzi* that were identified in 1977 by MLEE in Brazil.^1^ Brisse et al.,^5^ used a combination of MLST targets and confirmed the presence of six principal lineages (or distinct taxonomic units, DTUs), which corresponded with the six groups previously identified by MLEE. The six Brisse DTUs were initially named TcI and TcIIa to TcIIe. However, the initial MLST nomenclature of Brisse et al. did not appropriately represent the relationship between the six lineages. Therefore, TcI to TcVI was suggested as an alternative, with TcV and TcVI recognised as hybrids of TcII and TcIII.^6^ This nomenclature was adopted in the 2009 revision meeting organised by Zingales, as cited in the current review. Recently, a seventh DTU has been proposed, similar to TcI. It is conceivable that more DTUs remain to be discovered.

*Progress* - The ability to genotype isolates of *T. cruzi* by MLST, and by more complex DNA sequencing, has had multiple positive outcomes, for example:

(i) Resolving *T. cruzi* transmission pathways to guide Chagas disease control strategies, such as determining whether domestic and sylvatic transmission is overlapping, or non-overlapping and thus easier to control.^7^

(ii) Tracking the source of outbreaks transmitted orally.

(iii) Identifying DTUs in migrants from different endemic regions.

(iv) Comparing clinical presentation of chronic Chagas associated with different DTU infections.

(v) Identifying high risk triatomine vectors of *T. cruzi*, as done in enzootic analysis of transmission in the Amazon region.

(vi) Discovery of reservoir hosts.

However, isolation of *T. cruzi* from patients or reservoir hosts can be complex: organisms sequestered in the tissues can be distinct from those in the blood. Lineage-specific serological diagnosis provides one potential solution, which has shown that human TcII positive seroprevalence can be associated with more severe cardiomyopathy^8^ and that tamarins in the Atlantic Forest are reservoir hosts of TcV/V infection, not only of the TcII infection shown in Table IV of Zingales and Bartholomeu.^9^

The ability to sequence fully multiple genes of *T cruzi* or entire genomes, with a combination of Illumina and long read PacBio resolution, has given profound insight into phylogenetic relationships, genome structure and function. Historically it was proposed that trypanosomes and *Leishmania* were clonal, with genetic exchange having no significance for the epidemiology of *T. cruzi* infection*.* However, not only are two of the six *T. cruzi* lineages hybrids, in 2003 genetic exchange of *T. cruzi* was demonstrated *in vitro*, evidently with a mechanism via fusion of diploids. This has now been reaffirmed by detailed differential comparative genomics of housekeeping genes and multiple gene families.^10^ There is also strong evidence of meiotic genetic exchange of *T. cruzi* in natural populations.

As described in the Zingales and Bartholomeu review, there are multiple factors that influence immune response to *T. cruzi* infection and the parasite’s ability to survive lifelong in the host, unless successfully treated by chemotherapy. The extraordinarily complex multiple gene families, associated with retrotransposons and encoding surface proteins, implies that this diversity has a fundamental role in immune evasion, which is not yet fully understood.^11^

The ability to make transgenic *T. cruzi* carrying bioluminescent markers has transformed the efficiency of drug discovery and evaluation. Focal distribution of *T. cruzi* infections and drug efficacy can be monitored *in vivo* throughout the lifespan of individual mice. High resolution post-mortem confocal microscopy has revealed sequestration of infection in the intestinal tract, resurgence, and associated pathology.^12^

*Challenges* - The most magnificent achievement in combating Chagas disease has been the Southern Cone Initiative to control *Triatoma infestans*, led by Joao Carlos Pinto Dias, contributing substantially to the reduction of prevalence of infection from an estimated 18 million to approximately 7 million, and demonstrating that ‘disease control has no frontiers’.

However, several challenges remain. Despite intensive efforts and significant progress, Chagas disease remains a public health issue in the Chaco region of South America. It can also be resurgent and expansive in other endemic regions where control is not sustained. Insecticide resistance is emerging in triatomine bugs. With international migration, Chagas disease has also become a health issue in non-endemic regions.

Chemotherapy is still dependent on benznidazole or nifurtimox, although paediatric doses and shorter periods of benznidazole treatment have been introduced, and the Drugs for Neglected Diseases *initiative* (DNDi) continues to search for alternative drugs. As described in the Zingales and Bartholomeu review, although understanding of immune responses and genomic analyses have progressed, immune evasion is still not fully understood. Diagnosis of congenital infection requires improvement. There are no point-of-care early biomarkers of cure. Cost-effective vaccination remains a remote prospect.

Thus, despite the extent of interest in Chagas disease and the proliferation of research publications, more remains to be done.

There were many contributors to research mentioned here, including: Aluisio Prata, Philip Marsden, João Carlos Pinto Dias, Chris Schofield, Marinete Povoa, Ralph Lainson, Alejandro Luquetti, colleagues at LSHTM, the Instituto Evandro Chagas and the Wellcome Trust.

Comments on the article: Zingales B, Bartholomeu DC. *Trypanosoma cruzi* genetic diversity: impact on transmission cycles and Chagas disease. Mem Inst Oswaldo Cruz. 2021; e210193
